# Delayed Diagnosis of Osteogenesis Imperfecta: A Differential Diagnosis Guided by Competing Ocular Findings and a Lack of Family History

**DOI:** 10.7759/cureus.25822

**Published:** 2022-06-10

**Authors:** Eric B Nguyen, Erica Y Kim, Michelle I Malwane, Sergio Trejo, José R Cucalón-Calderón

**Affiliations:** 1 Pediatrics, University of Nevada Reno School of Medicine, Reno, USA

**Keywords:** medical interpretation, interpreter services, health care literacy, language barrier, kyphoscoliosis, low-trauma fractures, complexion-associated melanosis, osteoporosis, osteogenesis imperfecta

## Abstract

Osteogenesis imperfecta (OI) is an inherited connective tissue disorder with variable clinical manifestations involving many structures and organ systems, leading to characteristic presentations such as low bone mineral density (BMD), vertebral compression fractures, hearing loss, and blue sclerae. Even within the same family, individuals with the same inherited genotype may have differential presentations due to variable expressivity. Early diagnosis of OI in the pediatric population may allow for earlier treatment and interprofessional interventions. This case describes a minority female infant who initially presented with bilateral complexion-associated melanosis (CAM) inclusions in her eyes. The appearance of her inclusions was reminiscent of the blue sclera seen in OI; however, there was no clinical suspicion for OI on birth, developmental, and family histories. Her growth and development were unremarkable at all well-child checks until her three-year well-child check. It was then discovered that she suffered multiple long bone fractures due to low trauma, vertebral compression fractures, and kyphoscoliosis. Due to the occurrence of these fragility fractures, she was given a clinical diagnosis of osteoporosis with pending genetic testing for osteogenesis imperfecta. It was later discovered that there was, in fact, an extensive history of recurrent childhood fractures in the patient’s brother, mother, and numerous maternal relatives. Our case demonstrates the greater need for certified medical interpretation services to obtain clear past medical and family history, especially in the face of language barriers and low health literacy, in conjunction with clinical findings, i.e., CAM, to guide the differential diagnosis and subsequent management appropriately.

## Introduction

Osteogenesis imperfecta (OI) is an inherited connective tissue disorder affecting the synthesis of type I collagen with an estimated prevalence of 25,000-50,000 cases in the United States [1]. The pathogenesis of OI can be simplified into mutations of genes that control type I collagen synthesis or those that encode the proteins involved in the post-translational modification of type I collagen. It has been shown that those with defects in type I collagen synthesis possess either an alpha1 (COL1A1) or alpha2 (COL1A2) collagen type I defect [2]. Moreover, these mutations have been observed to exhibit Mendelian inheritance in an autosomal dominant manner. Overall, type I collagen synthesis defects represent the majority of OI cases, and variations in mutations of COL1A1 or COL1A2 are responsible for OI types I-IV [2]. 

Collagen is the proteinaceous basis of the extracellular matrix of all organs and tissues, making it the most abundant protein in the human body [3]. Accordingly, variant mutations in osteogenesis imperfecta can have significant pathologic manifestations throughout the body. Osteologic manifestations include low bone mineral density (BMD), fractures secondary to low trauma, hearing loss, vertebral compression fractures, scoliosis, and short stature, among many others. Extraskeletal manifestations include blue sclerae, dentinogenesis imperfecta, and hydrocephalus [2]. During the normal biosynthesis of collagen, post-translational modification of pro-alpha chains leads to the formation of a triple-helical association of three pro-alpha chains. It is the proper association of these three chains that forms procollagen, allowing it to be incorporated into tissues with subsequent modifications. The mutations involved in OI prevent the proper association of this basic structure of the collagen molecule, leading to the downstream phenotype [2].

Because of the variable presentation of OI and the possibility to achieve an early diagnosis in the pediatric population, it is crucial to combine clear and specific family history with clinical presentation to diagnose early, begin treatment, reduce the risk of complications, and improve patient outcomes. This case characterizes a patient with a delayed diagnosis of OI who presented initially with complexion-associated melanosis (CAM) and no reported close family history. She later presented with three long bone fractures in the span of five months, now with a family history significant for childhood fractures in her brother, mother, and a formal diagnosis of OI in a maternal aunt.

## Case presentation

This patient established care with our pediatrics clinic as a newborn from an affiliate hospital. The patient was born term at 39 weeks and four days by normal spontaneous vaginal delivery following an uncomplicated gestation. There was no birth trauma or history of fractures at the time of delivery. The patient’s mother herself sustained no fractures during birth. Additionally, no gestational or anatomic abnormalities were reported during obstetric visits. The patient followed up appropriately on all well-child visits.

At the nine-month well-child visit, the patient’s mother brought up the concern that she noticed blue discolorations in the patient’s eyes. Initially, she was diagnosed with bilateral melanosis oculi, a form of CAM, corroborated with similar-appearing inclusions noted in the mother’s eyes during the exam. Upon questioning, the patient’s mother reported that her maternal aunt was diagnosed with OI in Mexico; however, the family history excluded OI from all close family members, including parents and other siblings. The patient’s mother herself denied a history of blue eyes or inclusions, bone development problems, dental problems, or hearing loss. During this visit, bilateral leukocoria was also noted on the physical exam, and a referral to ophthalmology was made for both findings. Ophthalmology attributed the leukocoria to mild myopia and also agreed with the diagnosis of melanosis oculi. The patient continued well-child follow-up visits at 12, 15, and 18 months without any abnormal findings or concerns. Throughout all well-child visits, she has been tracking along at appropriate length and weight percentiles without signs of growth deceleration or stagnation.

**Figure 1:**
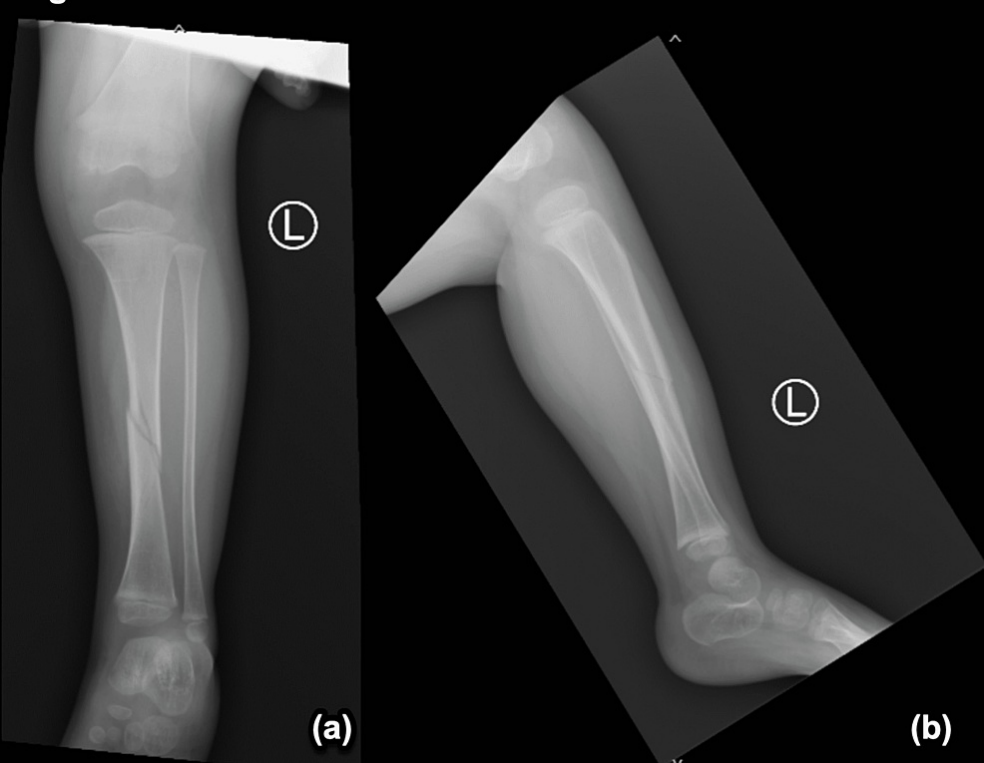
Oblique spiral midshaft diaphyseal fracture of the left tibia with (a) AP and (b) lateral views

**Figure 2:**
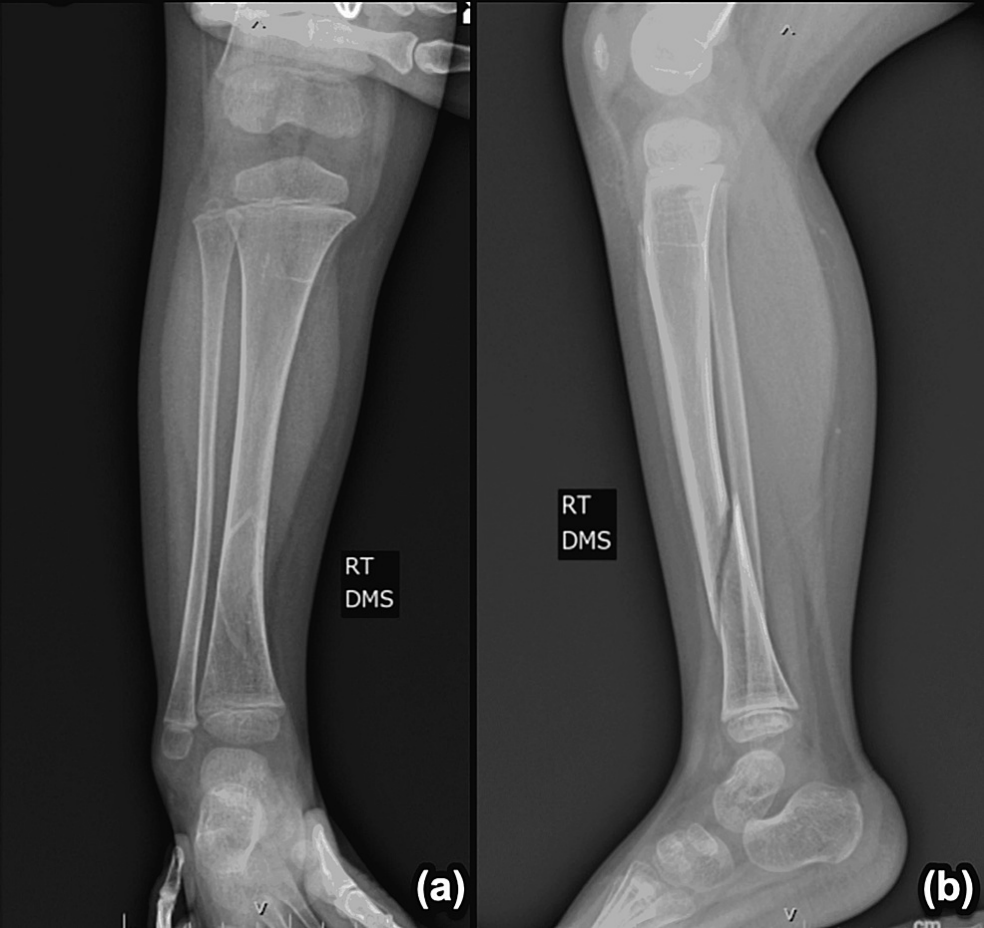
Right oblique tibial fracture with (a) AP and (b) lateral views

**Figure 3:**
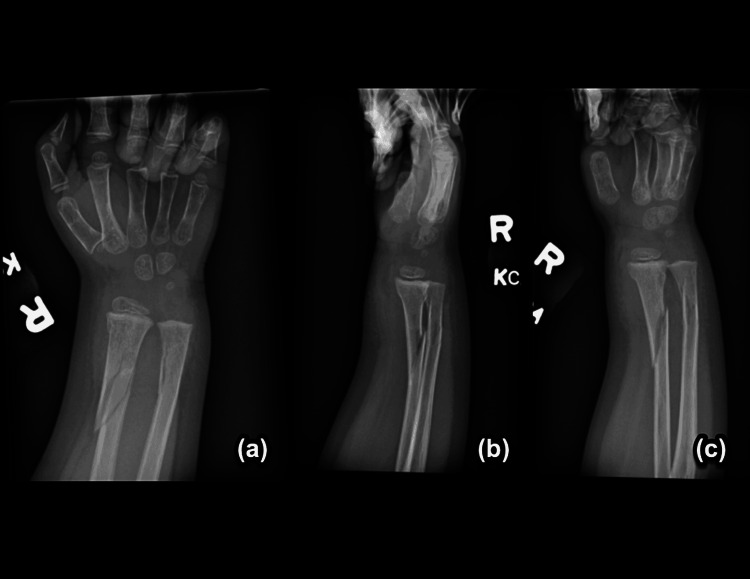
Right forearm fracture with (a) AP, (b) oblique, and (c) lateral views

**Figure 4:**
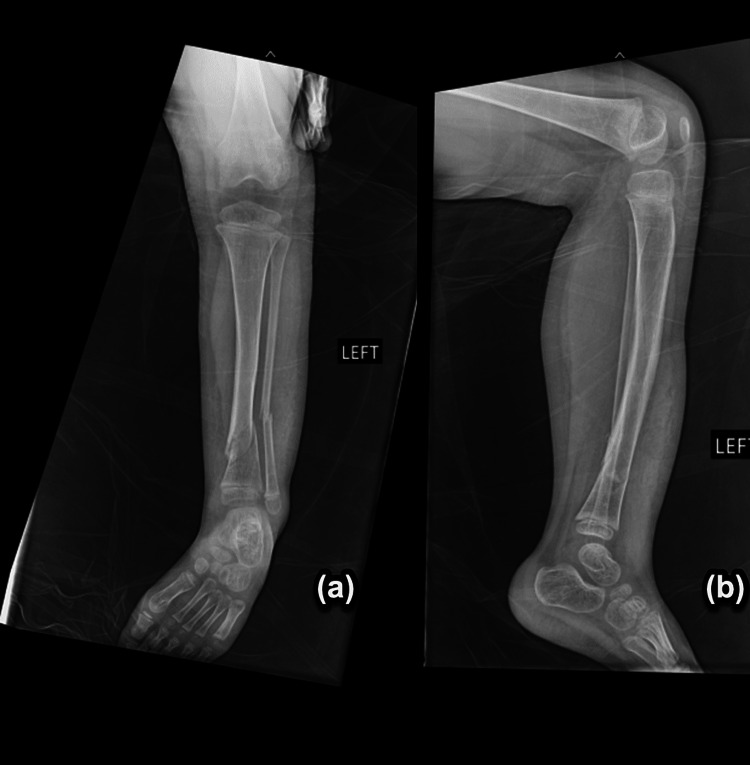
Comminuted fracture of the left distal tibial diametaphysis with (a) AP and (b) lateral views

The patient sustained her first fracture at 20 months of age, between her 18-month follow-up and her two-year follow-up (Figure 1a-1b). A radiology report in the emergency department (ED) denoted an oblique spiral midshaft diaphyseal fracture of the left tibia. Per the ED physician's notes, the patient’s father reported that she fell in the kitchen, landing on her left leg. Her second fracture occurred five months later, at two years of age. She sustained a right oblique tibial fracture due to a fall (Figure 2a-2b). After another five months, she suffered her third fracture, a fracture of the right forearm. X-ray imaging for this fracture was obtained; however, outside radiology reports were not available (Figure 3a-3c). Her fourth occurred approximately eight months later, at two years and nine months of age. She sustained a comminuted fracture of the left distal tibial diametaphysis due to a fall (Figure 4a-4b).

**Figure 5:**
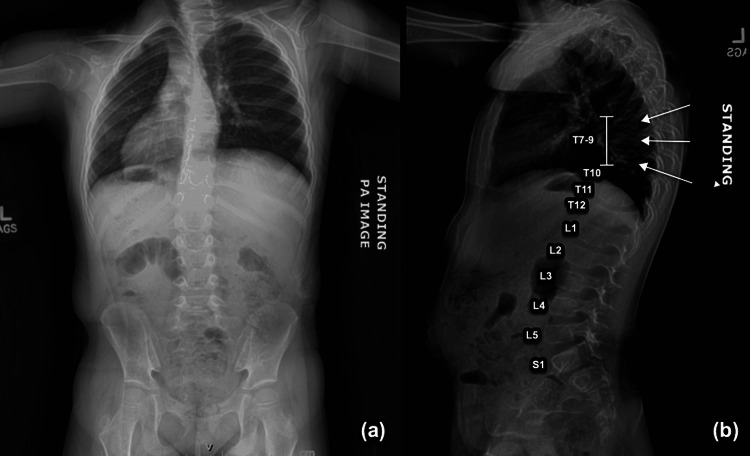
Scoliosis study denoting dextroscoliosis of the thoracic spine, kyphoscoliosis, and compression deformities at the T7-T9 levels with (a) PA and (b) lateral views

The patient did not attend her 30-month well-child visit and instead appeared for her three-year well-child visit after these four fractures had occurred. The patient was then referred to pediatric endocrinology for clinical suspicion of osteogenesis imperfecta. Given her history of fragility fractures, she was given a clinical diagnosis of osteoporosis. Dual X-ray energy absorptiometry (DEXA) determination of BMD was not obtained due to the unavailability of DEXA scans for pediatric patients in our state. The patient was also referred to pediatric orthopedics, who confirmed that the fractures were likely due to low trauma. An X-ray at the time of her orthopedic visit also revealed kyphosis with compression fractures of T7-T9 (Figure 5a-5b).

Given her clinical osteoporosis, evidence of thoracic compression fractures with kyphosis, and history of multiple long bone fractures secondary to low trauma, her history is suggestive of osteogenesis imperfecta type I. She was referred for genetic testing. Labs prior to administration of zoledronate for treatment of her osteoporosis, including CMP, serum calcium, 25-hydroxyvitamin D, 1,25-hydroxyvitamin D, and PTH, were all within normal limits. At the time of writing, genetic testing is still pending to evaluate for a definitive diagnosis of osteogenesis imperfecta. 

## Discussion

OI is a relatively rare disorder that is important to diagnose early due to its variable presentation and numerous manifestations in pediatric patients that may require multiple treatment modalities for the disease sequelae. Early recognition and diagnosis of osteogenesis imperfecta can help parents be aware of the clinical features of the disorder and assist in developing support systems for patients as they continue through growth and development. This is especially true for the more common, yet milder, forms of OI that may not be screened for and/or detected as readily as the more severe forms. Moreover, although genetic testing is becoming more readily available, the strong clinical suspicion needed to pursue further testing must come from a thorough history, including past medical, family, and social history, in addition to a careful physical exam.

This patient’s initial heralding sign of OI was her ambiguous conjunctival/scleral abnormalities noted on the physical exam for her nine-month well-child visit. Initially, the differential for these discolorations included complexion-associated melanosis or blue sclera secondary to osteogenesis imperfecta. CAM presents as flat, noncystic brown pigmentation of the conjunctiva, especially in patients with darker complexions [4]. However, the pigmentation can often range from slate-gray to bluish in color, such as in our patient, making it difficult to narrow the differential [5].

With a negative family history at the time, similar discoloration in the mother’s eyes, and the relative rarity of OI, the presence of blue sclera was not fully explained by a diagnosis of OI, rendering the findings more clinically consistent with CAM. Accordingly, there was not a high enough pretest probability to order genetic testing to assess for OI. On initial presentation to our clinic as a newborn, the patient had also not experienced any fractures, dentinogenesis imperfecta, or growth delay, further lowering the clinical suspicion and need for genetic testing. 

Moreover, language and cultural barriers, in addition low health literacy, likely contributed to difficulty in obtaining a reliable family history in this patient. The patient’s family is Spanish-speaking, and Spanish interpretation services, provided by our affiliate hospital, were used during all visits. OI was a key differential diagnosis to rule out not only because of its similar scleral manifestations in our patient but specifically because the patient’s mother disclosed a history of OI in the patient’s maternal aunt. Accordingly, we performed a focused family history, with questions focused on the clinical features of OI in close relatives to assess inheritance patterns, e.g., recurrent childhood fractures, dentinogenesis imperfecta, birth trauma, etc. The patient’s mother initially denied the presence of any clinical features of OI in the family. However, upon further history gathering at later visits, the patient’s mother reported that she herself had a similar history of multiple fractures in childhood. Additionally, the patient’s older brother, who follows with a different pediatrician, had sustained over seven low-trauma fractures until age 10. No workup nor further evaluation has been done for the brother. It was then disclosed that the patient’s maternal uncle, grandmother, and great uncle all had histories of recurrent fractures in childhood. 

It is not entirely clear what prompted the change in history from one encounter to the next; however, we suspect a potential reason for miscommunication is a lack of certified medical Spanish interpretation services in our state and broadly throughout the United States. Medical interpreters in the United States are certified by the National Board of Certification for Medical Interpreters (NBCMI) and by the Certification Commission for Healthcare Interpreters (CCHI), both of which were established in 2009 to verify interpreter training and to protect patient safety [6]. The NBCMI maintains a standardized process for interpreter certification. Those seeking certification must demonstrate proof of oral proficiency in both English and Spanish, e.g., by possessing degrees of higher education in both respective languages or demonstrating passing marks on standardized language proficiency exams, such as the Test of English as a Foreign Language (TOEFL) and the American Council on the Teaching of Foreign Languages (ACTFL) Oral Exams. They must also complete a minimum of 40 hours of training in medical interpretation and pass both written and oral exams administered by the NBCMI [6]. CCHI certification has similar requirements and examinations to interpreter certification [6]. Because of the demanding nature of these requirements for certification, at the time of writing, there are only 6,948 certified medical Spanish interpreters in the United States listed in the NBCMI and CCHI registries [7,8]. However, according to the US Census Bureau’s 2016-2020 American Community Survey five-year estimate, 13.2% of the population speaks Spanish, and 39.3% of these Spanish speakers reported that they speak English less than "very well," representing around 16 million people [9]. This clear disparity in the supply of certified medical interpreters leads to the use of uncertified medical interpreters, ad hoc interpreters, e.g., family members, bilingual children, friends, untrained staff members, etc., or no interpreter use altogether [10]. A cross-sectional study performed by Flores et al. analyzed audio recordings and transcripts of Spanish clinical encounters in which professional interpreters, ad hoc interpreters, or no interpreters were present. The study demonstrated that the proportion of errors of potential clinical consequence, i.e., interpretation errors leading to changes in diagnostic reasoning, management, or patient education, was significantly lower for professional interpreters (12%) versus ad hoc (22%), or having no interpreters (20%) [10]. This case reflects an increased need to support training for certified medical interpreters since the evidence has shown better patient outcomes with the use of these services [10].

In addition to language barriers, we suspect that factors of low health literacy contributed to incomplete family history. This is namely from the fact that the patient’s mother did not mention her extensive family history of fractures despite having been asked directly. Therefore, it is unlikely that she understood the hereditary nature of the diagnosis in question, especially since she explicitly reported a history of OI in her sister, i.e., the patient’s maternal aunt, without drawing a connection to her other relatives. This is further corroborated by the fact that it was ultimately discovered that her son, the patient’s brother, had a very similar clinical presentation to the patient that was not mentioned until later visits. Ultimately, this case reflects inadequate physician-patient education and/or a failure to provide appropriate education in the setting of lower health literacy. Physician-patient education can be improved for patients with low health literacy by not only ensuring appropriate interpretation services but also by adapting a culturally sensitive approach. This can entail simplifying medical language, practicing spaced repetition of important information across multiple visits, and providing both intelligible written and oral instructions [11]. 

While it is important to consider the factors that contributed to the delayed diagnosis of OI in this patient, it is equally important to discuss the implications of early treatment. The current standard of treatment for OI is adequate dietary supplementation of vitamin D and calcium in addition to bisphosphonates, which inhibit bone resorption. The current data suggest that treatment with IV or oral bisphosphonates, i.e., zoledronate, pamidronate, neridronate, etc., increases BMD in patients with OI compared to placebo. However, it is unclear whether the increase in BMD correlates with actual clinical improvement [12]. A systematic review by Dwan et al. in 2016 reported that of the 14 studies assessing changes in fracture incidence on bisphosphonate therapy, none resulted in an increased fracture incidence, with one study demonstrating a reduction in relative risk of fracture reaching as high as 31% with a hazard ratio of 0.69 (95% CI 0.52-0.91) [12,13]. Long-term data on the safety of bisphosphonate drugs in children is still unclear and is an area of active research, but the benefits may outweigh risks for many patients, especially for patients with severe OI, e.g., OI type III. This is supported by a study from Antoniazzi et al. demonstrating that early neridronate treatment resulted in a decrease in fracture rate for those who started treatment just after birth compared to those who started treatment after six months and those in the placebo group (2.4 vs. 6.0 and 6.8 fractures/year, respectively; p<0.5) [14].

Earlier diagnosis in this patient would have permitted starting physical therapy and interprofessional interventions sooner. However, starting these interventions in this patient is already challenging at baseline, given that the patient’s family depends on Medicaid for healthcare services. Other complicating socioeconomic determinants of health, including income, language barriers, and level of education, may make it more difficult to find, finance, and travel to office visits, diagnostic testing, and infusion services. Moreover, many services, such as genetic counseling and DEXA scans for pediatric patients, are not readily available in our state, which incurs additional costs of travel out of state for these services, all of which may further delay the time to definitive management of the disorder. All of these additional factors further emphasize the importance of prompt diagnosis to ensure that patients and their families have the time and resources to pursue appropriate treatment.

## Conclusions

OI is a rare genetic disorder of collagen formation characterized by poor bone mineralization, frequent fractures, and numerous extraskeletal manifestations. This can increase the risk of many developmental abnormalities in pediatric patients, necessitating the services of an interprofessional group of physicians and healthcare providers to manage subsequent comorbidities and facilitate normal growth and development. Although more research is needed, a key component to managing OI is early diagnosis and treatment, especially when individual factors, such as incomplete family history or socioeconomic factors, can delay diagnosis and treatment, leading to adverse health outcomes.
